# Handoffs and transitions in critical care—understanding scalability: study protocol for a multicenter stepped wedge type 2 hybrid effectiveness-implementation trial

**DOI:** 10.1186/s13012-021-01131-1

**Published:** 2021-06-15

**Authors:** Meghan B. Lane-Fall, Athena Christakos, Gina C. Russell, Bat-Zion Hose, Elizabeth D. Dauer, Philip E. Greilich, Bommy Hong Mershon, Christopher P. Potestio, Erin W. Pukenas, John R. Kimberly, Alisa J. Stephens-Shields, Rebecca L. Trotta, Rinad S. Beidas, Ellen J. Bass

**Affiliations:** 1 423 Guardian Drive, 309 Blockley Hall, Philadelphia, PA 19104 USA; 2 3400 Spruce Street 6th Floor Dulles Building, Philadelphia, PA 19104 USA; 3 3400 Civic Center Boulevard, Building 421, Philadelphia, PA 19104 USA; 4 423 Guardian Drive, 333 Blockley Hall, Philadelphia, PA 19104 USA; 5 2450 W Hunting Park Ave, 3rd floor, Philadelphia, PA 19129 USA; 6 5323 Harry Hines Blvd, Dallas, TX 75930 USA; 7 1800 Orleans St, Suite 6364, Baltimore, MD 21287 USA; 8 1 Cooper Plaza, Camden, NJ 08103 USA; 9 401 S. Broadway, Camden, NJ 08103 USA; 10 3620 Locust Walk, 2109 Steinberg-Dietrich Hall, Philadelphia, PA 19104 USA; 11 423 Guardian Drive, 628 Blockley Hall, Philadelphia, PA 19104 USA; 12 3400 Spruce St., Philadelphia, PA 19104 USA; 13 3535 Market Street, Ste 3105, Philadelphia, PA 19104 USA; 14 Drexel University, 3675 Market Street, Suite 1000, Philadelphia, PA 19104 USA

**Keywords:** Critical care, Ergonomics, Evidence-based practice, Human factors engineering, Implementation science, Hybrid effectiveness-implementation trials, Medical communication, Patient safety, Patient handoff, Postoperative period, Transition of care

## Abstract

**Background:**

The implementation of evidence-based practices in critical care faces specific challenges, including intense time pressure and patient acuity. These challenges result in evidence-to-practice gaps that diminish the impact of proven-effective interventions for patients requiring intensive care unit support. Research is needed to understand and address implementation determinants in critical care settings.

**Methods:**

The Handoffs and Transitions in Critical Care—Understanding Scalability (HATRICC-US) study is a Type 2 hybrid effectiveness-implementation trial of standardized operating room (OR) to intensive care unit (ICU) handoffs. This mixed methods study will use a stepped wedge design with randomized roll out to test the effectiveness of a customized protocol for structuring communication between clinicians in the OR and the ICU. The study will be conducted in twelve ICUs (10 adult, 2 pediatric) based in five United States academic health systems. Contextual inquiry incorporating implementation science, systems engineering, and human factors engineering approaches will guide both protocol customization and identification of protocol implementation determinants. Implementation mapping will be used to select appropriate implementation strategies for each setting. Human-centered design will be used to create a digital toolkit for dissemination of study findings. The primary implementation outcome will be fidelity to the customized handoff protocol (unit of analysis: handoff). The primary effectiveness outcome will be a composite measure of new-onset organ failure cases (unit of analysis: ICU).

**Discussion:**

The HATRICC-US study will customize, implement, and evaluate standardized procedures for OR to ICU handoffs in a heterogenous group of United States academic medical center intensive care units. Findings from this study have the potential to improve postsurgical communication, decrease adverse clinical outcomes, and inform the implementation of other evidence-based practices in critical care settings.

**Trial registration:**

ClinicalTrials.gov identifier: NCT04571749. Date of registration: October 1, 2020.

**Supplementary Information:**

The online version contains supplementary material available at 10.1186/s13012-021-01131-1.

Contributions to the literature

- This protocol provides a structured methodology for understanding barriers to implementation to evidence-based practices in critical care settings, namely the time pressure experienced by clinicians and the complexities inherent in high patient acuity.
- This protocol highlights the utility of combining implementation science, systems engineering, and human factors engineering to understand and implement interventions that rely upon complex workflow patterns.
- Our prior work in two hospitals demonstrated the feasibility and acceptability of standardizing handoff communication for patients requiring intensive care after surgery by use of a protocol with defined clinician roles and responsibilities.
- The HATRICC-US study will expand the scope of our prior work, testing both the implementation and effectiveness of standardizing operating room to intensive care unit handoffs in five academic health systems in the United States.

## Background

Multiple challenges prevent the optimal implementation of evidence-based practices (EBPs) in the intensive care unit (ICU), including heterogeneity in evidence quality, clinician staffing, team structures and workflow, as well as time pressure inherent in caring for critically ill patients [1]. These challenges complicate the study of ICU-based implementation efforts, which are under-represented in the implementation science literature [2]. As a result, there are critical knowledge gaps regarding how to facilitate the consistent adoption of EBPs in the ICU. These knowledge gaps may partially explain the underutilization of critical care EBPs that contributes to suboptimal patient outcomes [3].

Many EBPs in the ICU are meant to guide the complex workflow of the critical care team. These include structured multidisciplinary rounds [4–7], the use of protocols to facilitate weaning from mechanical ventilation [8–10], and the adoption of structured handoff protocols for patient transfer from the operating room (OR) to the ICU [11–14]. When employed, these EBPs improve patient outcomes through, for example, the prevention of medical errors and adverse events [15, 16]. In practice however, they are incompletely adopted [17–19].

Of these interventions, OR to ICU handoff standardization is a frequent topic of study; a recent systematic review and meta-analysis found 32 OR to ICU handoff intervention studies published since 2007, 24 of which were published in 2015 or later [20]. These handoffs are frequent events involving the exchange of relevant information about a patient’s history and perioperative course and the explicit transfer of patient care accountability from surgical to ICU teams [20]. These procedures involve clinicians from different disciplines (i.e., surgery, anesthesia, and intensive care), and patients who are frequently unable to participate in their care at the time of handoff. Handoffs are complex sociotechnical interactions that depend on the specific internal and external aspects of each workplace. Thus, any adaptation of the handoff process presents the added implementation barrier of needing to be tailored to the settings in which these handoffs occur.

In the Handoffs and Transitions in Critical Care (HATRICC) study, we studied the effectiveness and implementation of a standardized OR to ICU handoff protocol in mixed surgical ICUs [12–14, 21–23]. From an implementation perspective, barriers to achieving a standardized handoff process included unclear expectations, time pressure, and confusion about other providers’ needs [13]. Multiple stakeholder perspectives provided important contextual information used as inputs for the selection of implementation strategies to support the uptake and use of a new handoff protocol. We combined findings from the contextual inquiry, a synthesis of pre-existing literature, and in situ simulation to design a standardized handoff protocol. We showed that handoffs adhering to this protocol improved information exchange and had high clinician acceptability [13, 14]. Although this work was one of the larger studies of OR to ICU handoffs [20], it was effectively a pilot study limited in detecting associations between the intervention and clinical outcomes due to its modest sample size (*n* = 165 observations). Implementation lessons were similarly limited given the scope of the study, which was based in two ICUs in two hospitals in a single health system.

The present study builds on this prior work by scaling up to multiple academic health systems. This fully powered trial will allow us to test confirmatory hypotheses and extend our understanding to more health systems. We will therefore be able to characterize implementation determinants in multiple centers and will be able to test relationships between the intervention and patient outcomes. This study may also allow us to characterize implementation determinants in critical care more broadly. The HATRICC handoff protocol serves as an exemplar use case for testing implementation in critical care, as OR-to-ICU handoffs are common (> 10 handoffs per ICU per week), occur in a known location, and are a complex interaction requiring engagement from multiple care team members.

## Methods/design

This manuscript adheres to the Standards for Reporting Implementation Studies (StaRI) Statement [24] (Additional file 1: Appendix).

HATRICC-US is a type 2 hybrid effectiveness-implementation study [25] with a convergent mixed methods (QUAL + QUAN) [26] approach. The study is quasi-experimental in design, using a stepped wedge design with randomized roll-out [27]. Study recruitment started on March 24, 2021. Data collection started in April 2021 with elicitation interviews; handoff observations are scheduled to begin in July 2021.

### Regulatory considerations

HATRICC-US was registered on ClinicalTrials.gov on October 1, 2020 (NCT04571749). A single institutional review board (IRB) based at the University of Pennsylvania is the IRB of record; this IRB reviewed and approved the study on November 10, 2020 (#843670). Reliance agreements were completed by the participating institutions and are managed using the SMART-IRB (Streamlined, Multisite, Accelerated Resources for Trials IRB Reliance) platform [28]. The research was deemed minimal risk, so no data safety and monitoring board was convened. The research is guided by an external advisory board consisting of four experts in implementation science, human factors engineering, clinical research, and perioperative clinical care, respectively. The advisory board will meet two times annually for the duration of the study and had met once at the time of the writing of this manuscript.

### The process to be studied: OR-to-ICU handoffs

**Fig. 1:**
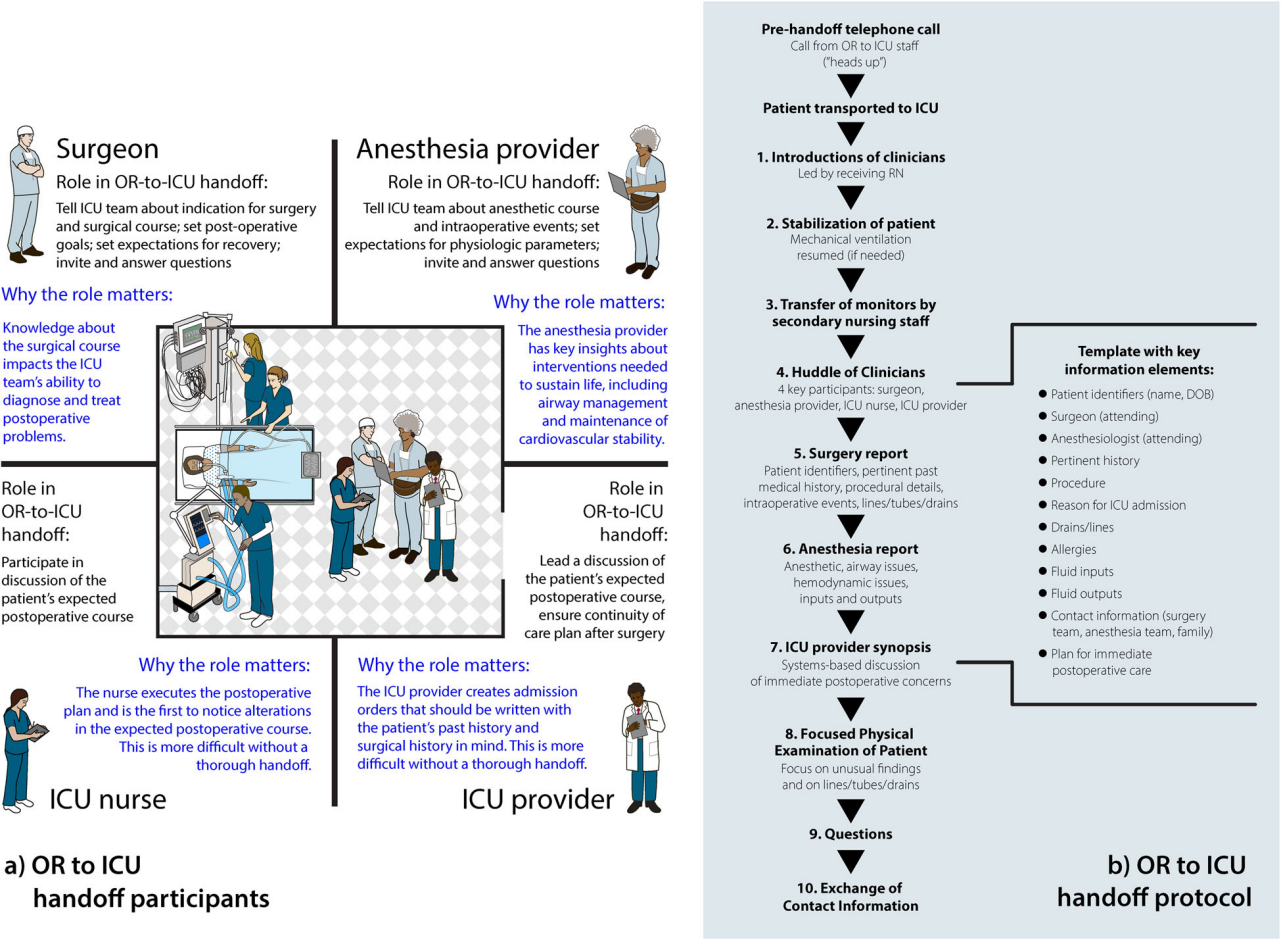
**a** Clinician participants and roles in the OR-to-ICU handoff. **b** OR-to-ICU handoff protocol

An example of the OR to ICU handoff process is illustrated in Fig. 1. The handoff process generally unfolds as follows: Before or during surgery, a given patient is identified as requiring post-surgical ICU admission. The ICU charge nurse is notified either by electronic health record tracking systems or by telephone of the patient’s impending admission. At the conclusion of the surgical procedure, the patient is either (1) transported directly from the OR to the ICU by members of the surgical and anesthesia teams or (2) transported to a post-anesthesia care unit by the surgical and anesthesia teams, then to the intensive care unit by a registered nurse and patient transporter. During transport to the ICU, the patient’s vital signs are continuously observed with electronic transport monitors that display blood pressure, heart rate, and oxygen saturation by pulse oximetry. If necessary, mechanical ventilation is applied and resuscitative medications are administered. On arrival to the ICU, physiologic monitors are transferred to in-room equipment. Shortly after the patient arrives in the ICU, verbal and/or written communication between the surgery, anesthesia, and ICU teams occurs. Typically, this “handoff team” consists of a surgeon, a nurse anesthetist or physician anesthesiologist (hereafter called “anesthesia provider”), an ICU registered nurse, and an ICU ordering provider (physician, nurse practitioner, or physician assistant). The communication among these team members includes a discussion of the patient’s relevant history, intra-procedural details, and anticipatory guidance about postoperative care. Institutions differ in handoff timing (synchronous or asynchronous with respect to direct patient care), location of this handoff (at bedside or otherwise), and composition of the handoff team.

### The evidence-based practice: OR-to-ICU handoff protocol

**Fig. 2:**
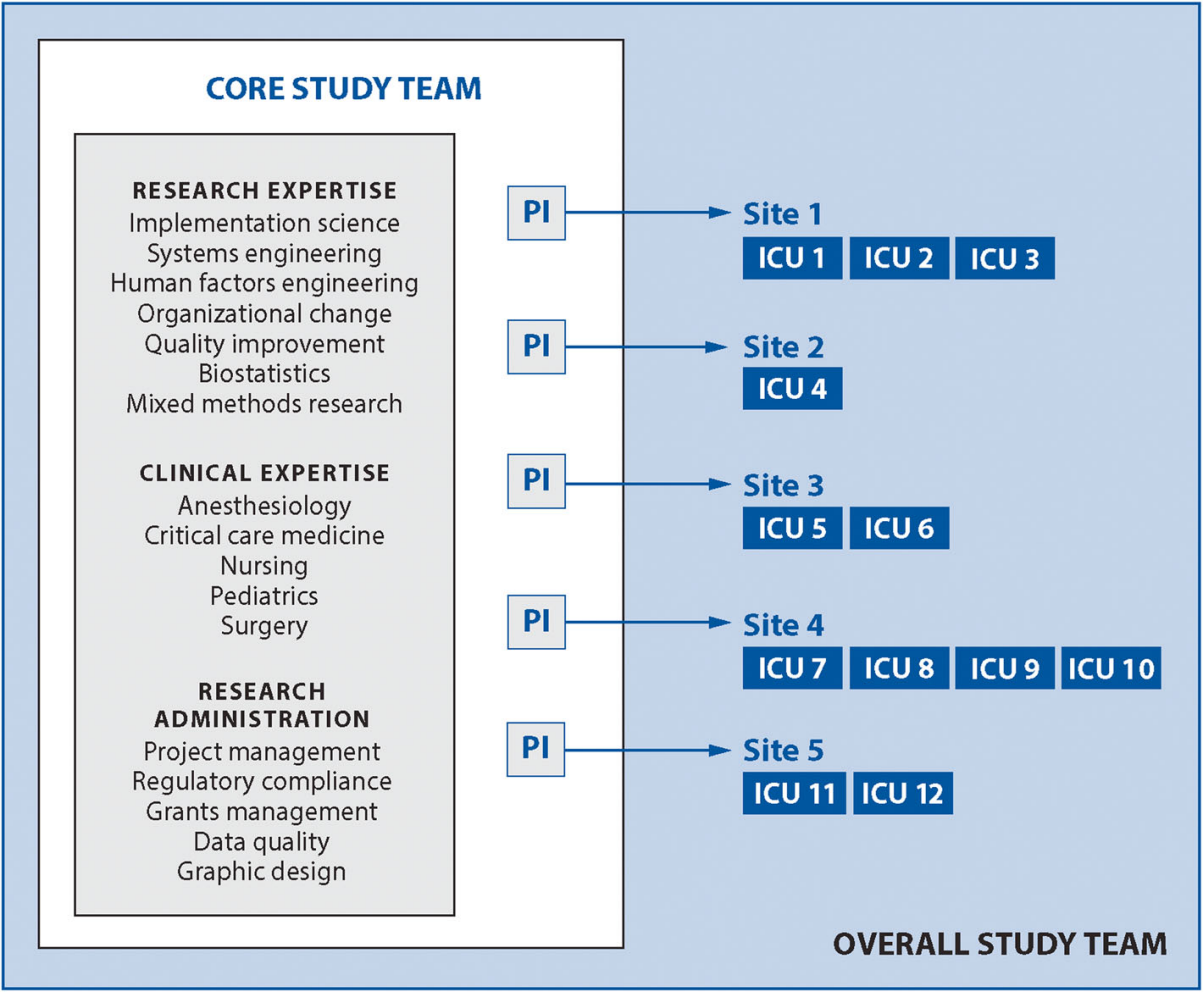
Study team structure and governance

Since 1990, at least 65 published studies have reported on the effects of standardizing these handoffs [11, 20, 29]; they demonstrated improved information exchange [14, 30] and patient outcomes [31–33] with standardization. As such, the American Heart Association has endorsed the use of structured protocols to guide perioperative clinician communication [34]. Published standardized handoff protocols include two core elements: (1) a face-to-face group conversation at the patient’s bedside (Fig. 1a), and (2) use of a template or checklist to structure that conversation (Fig. 1b) [23]. However, adoption and adherence to such protocols encounters challenges in clinical practice [35].

### Study team and governance

The study team is a multidisciplinary, multiprofessional group of researchers and subject matter experts with backgrounds in implementation science, human factors engineering, systems engineering, change management, organizational theory, biostatistics, mixed methods research, anesthesiology, critical care, nursing, and surgery. There are no patients or caregivers as part of the study team, as the process of interest for the current study concerns technical aspects of care that are in many ways invisible to patients and caregivers. The core research team consists of an overall Principal Investigator (PI), four additional site PIs, faculty-level collaborators, and research staff (Fig. 2). Each “site” is an academic health system contributing 1–4 ICUs to the overall study, for a total of 12 ICUs. Each ICU represents a cluster whose implementation start time will be randomized.

Site PIs partner with clinicians and clinical leaders in each ICU to form 4-person implementation teams representing each of the four clinician roles (surgeon, anesthesia provider, ICU nurse, ICU provider) in an OR to ICU handoff. Implementation teams will work with the core research team to customize and implement their ICU’s customized version of the HATRICC protocol. Implementation teams are supported with a modest stipend ($5000 per ICU/year) to facilitate participation in the project. The site PIs and implementation teams are permitted to expend the stipend in any way needed to support the project; example expenses include staff time and creation of implementation materials.

### Guiding implementation frameworks and models

**Fig. 3:**
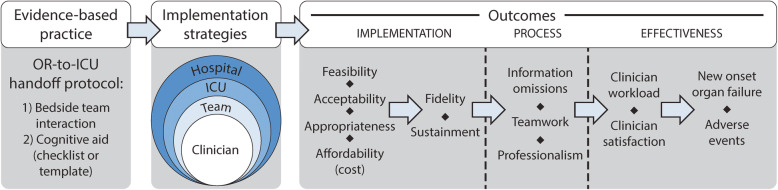
Hybrid of Proctor’s implementation model and the social ecological model

This study is guided by frameworks that represent the three major branches of Nilsen’s taxonomy, i.e., those that describe the implementation process, implementation determinants, and those that can be used for evaluation of the implementation effort [36]. We selected the EPIS (Exploration, Preparation, Implementation, and Sustainment) model by Aarons et al. [37] and the Tailored Implementation for Chronic Diseases (TICD) checklist [38] to guide contextual inquiry and overall study flow. We used a hybridization of Proctor’s framework [39] and the social ecological model [40] (Fig. 3) to demonstrate putative relationships between the implementation strategies to be used and the outcomes of interest. Finally, in recognition of the systems and human factors engineering principles relevant to the handoff process, we use the Systems Engineering Initiative for Patient Safety 3.0 model [41] to inform our approach to participatory design in the customization of handoff protocols for each ICU.

## Study aims and approach

### Aim #1: ascertain determinants of OR to ICU protocol adoption and use in 12 adult and pediatric ICUs in 5 health systems

Contextual inquiry will be used to achieve this aim. Site visits were initially planned but were canceled due to COVID-19 travel restrictions. In lieu of site visits, the core study team will conduct virtual interviews and focus groups using the Zoom platform (Zoom Video Communications, Inc, San Jose, CA) and will direct the completion of surveys and in-person observations to be conducted by on-site teams. We will use a convergent mixed methods approach [26], merging and connecting qualitative and quantitative datasets to develop a robust understanding of the environment in each ICU.

**Fig. 4:**
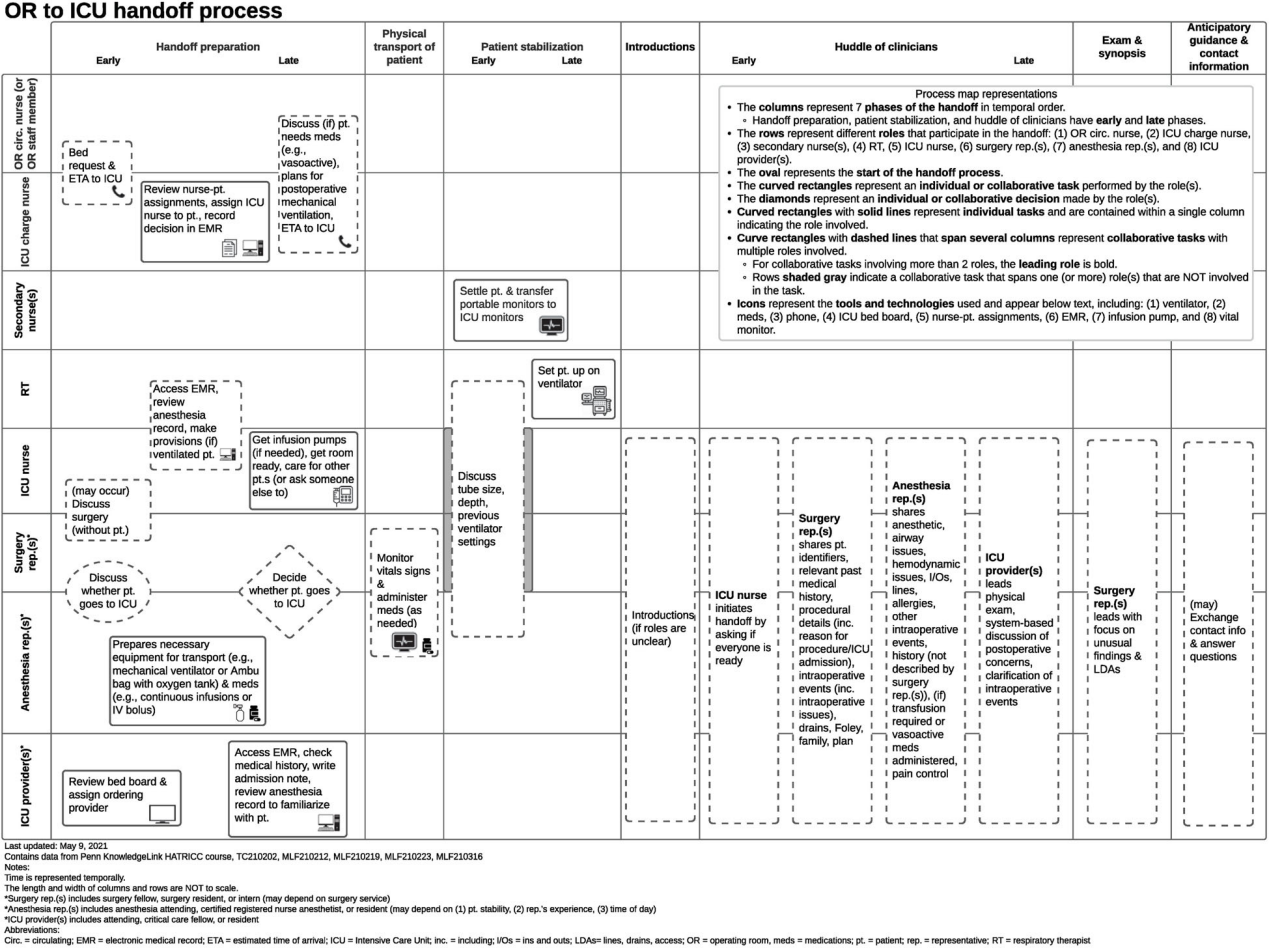
Exemplar process map of OR-to-ICU handoffs

Contextual inquiry will start with the development of individual process maps for the 12 ICUs using knowledge elicitation through virtual subject matter expert interviews according to the method described by Wooldridge et al. [42]. Process mapping uses human factors engineering approaches, e.g., semi-structured elicitation interviews with subject matter experts, to systematically evaluate elements of a complex sociotechnical work system in which a care process is embedded. This analysis of work system elements includes people (e.g., clinician stakeholders), technologies and tools, organization, and internal and external environment to both inform a representation of the process across time and physical location. Process maps can be represented visually in multiple ways [43]; we opted to use a swim lane diagram (Fig. 4) that shows a timeline of the handoff, visualizing the location, and relevant providers at each time point.

After process mapping, we will conduct another set of interviews and focus group discussions to identify handoff protocol implementation determinants (i.e., barriers to and facilitators of implementation) in each ICU. Clinicians representing the key roles in OR to ICU handoffs (surgeon, anesthesia provider, nurse, ICU ordering provider) will participate in mixed-role focus groups facilitated by trained members of the core study team. Participants will provide multiple perspectives on post-surgical handoffs, protocols, and clinical quality improvement initiatives. Given their smaller number, clinical leaders and administrators will participate in semi-structured interviews about their multiple perspectives on ICU protocols and then OR to ICU protocols specifically. Both focus group and interview scripts were designed for this study and informed by EPIS [37] and the TICD Checklist [38]; research team members and clinician subject matter experts selected relevant determinants from these frameworks. Participants will be recruited using purposive and snowball sampling [44]. Within each site, recruitment will be stratified by role, gender, and experience level.

Observations will be conducted by on site staff and will be directed by site PIs. Trained observers will use a common report form to capture details about clinical topics discussed during OR to ICU handoffs, clinicians present during each handoff, and fidelity, defined as adherence to the ten elements of the originally published HATRICC protocol (i.e., introduction of team members upon patient arrival, patient stabilization, transfer of patient to in-room monitors, huddle of handoff team members, surgery report, anesthesia report, ICU provider synthesis, team physical exam of patient, exchange of contact information, question asking period (Fig. 1b)) [14]. We expect fidelity at baseline to be greater than zero, as some of the protocol elements are commonly conducted even in the absence of a protocol.

We will use surveys as the quantitative element of the contextual inquiry, measuring clinician perspectives on OR to ICU handoffs using an adaptation of the survey we used in the original study [13] and perceived workload related to these handoffs using an adaptation of the NASA Task Load Index [45] (non-workload questions will be omitted). Surveys will allow us to incorporate the perspectives of more stakeholders than would be possible with interviews or focus groups alone.

### Aim #2: adapt handoff protocols using engineering approaches and select tailored implementation strategies with implementation mapping

Our experience with the original study suggests that OR to ICU handoff protocols must be compatible with the clinicians’ environment; they must complement or even improve clinicians’ workflow to be accepted and successfully implemented. Given that the workflow of each ICU is likely to be slightly different, we will create an adapted version of the HATRICC protocol to suit each ICU. What will remain consistent among all protocols are the “core elements” (face-to-face interaction, use of a written tool or template (Fig. 1)) and 10 basic steps identified in handoffs in the original HATRICC study [14].

To design ICU-specific versions of the HATRICC protocol, we will use an engineering approach known as *participatory design* [46, 47], which is distinct from other participatory methods such as community-based participatory research [48] or human/user-centered design [49]. Participatory design allows for users’ baseline knowledge of a process and workflow to assist in the customization of a new process. In this case, clinical care teams (i.e., surgeons, anesthesia providers, nurses, ICU providers) will be iteratively engaged in an interactive process generating multiple prototypes. We will use multiple methods to achieve engagement, including journey mapping [50] and brainstorming. Journey mapping will involve describing experiences with the current handoff process, including what is challenging about the current process and suggestions for unit-specific changes. Brainstorming is an opportunity for many different ideas to be generated quickly. This participatory design process will result in a final customized handoff protocol representing a composite that is co-created by representatives from all stakeholder groups. Cognitive aids for each protocol will be created with the assistance of a professional graphic designer.

We will synthesize our findings from aim 1 and from the participatory design sessions to develop a nuanced understanding of the barriers and facilitators of handoff protocol implementation that are common to all ICUs and those that are ICU specific. We will use implementation mapping [51] to select implementation strategies for each ICU, targeting those implementation determinants deemed most important and most addressable by site PIs and local implementation teams. Each ICU will also have access to 5 h of graphic designer time per year to create educational and other promotional materials. In addition to customizing the HATRICC protocol for each ICU, we anticipate selecting 1–3 additional implementation strategies per ICU tailored to each unit’s individual implementation determinants (e.g., coaching and empowerment for ICUs where nurses are afraid to speak up, or facilitating the relay of clinical information from the OR to the ICU via the electronic health record in ICUs where information transfer is unreliable).

### Aim #3: test the effectiveness of tailored, multifaceted, multilevel implementation strategies

After 4–6 weeks of baseline data collection in all ICUs, each ICU will implement the new HATRICC protocol using the implementation strategies selected in aim 2. We shall use a stepped wedge [52] approach to implementation start time, with each new “step” occurring 6–8 weeks after the prior step. The step duration was selected to balance the goal of clearly demarcating the change to a new handoff procedure and allowing for “wash in” (favors a longer step) with the goal of completing the implementation efforts in all ICUs with sufficient time to collect at least 12 months of sustainment data (favors a shorter step). This stepped wedge approach will enable each ICU to serve as its own control. Implementation start times will be randomized, though in hospitals with more than one ICU participating, ICU start times will be sequential. All ICUs will collect data for the duration of the study, estimated to be 4 years.

Aim 3 measures are shown in Table 1. The co-primary outcomes are fidelity (implementation outcome at the level of each handoff observation) and cases of new onset organ failure (effectiveness outcome at the level of the ICU).

**Table 1:** HATRICC-US outcome measures

Outcome (type)	Rationale	Unit of analysis and approach to measurement	Frequency and timing of measurement
Co-primary outcomes			
Fidelity (Imp)	Fidelity is a necessary precursor to effectiveness	Handoff-level; observations by site-based staff, count on a 10-point scale (quant), field notes (qual)	Monthly, Years 2-5
New-onset organ failure (Eff)	Per-protocol handoffs enable clinicians to follow expected care practices and to anticipate and avoid postoperative deterioration	ICU-level; composite measure of AHRQ Patient Safety Indicators (PSIs) [53] that reflect organ failure (quant)	Monthly, Years 2-5
Secondary outcomes			
Feasibility (Imp) Acceptability (Imp) Appropriateness (Imp)	These “early” implementation outcomes will influence subsequent fidelity and will help in the interpretation of fidelity findings	Clinician- and ICU-level; AIM [54], FIM [54], IAM [54] (quant); site visit findings (qual)	3 times: Year 1; within 2 months of implementation (Years 2-3); within 2 months of sustainment start (Years 4–5)
Sustainment (Imp)	Sustainment is the ultimate goal of the implementation effort	Handoff-level; characterized as fidelity over time (quant)	Monthly, Years 4–5
Affordability (Cost; Imp)	Implementation cost is an important consideration for transferability of study findings	ICU-level; accounting-based cost analysis as described by Hoeft et al. [55] (quant)	Within 2 months of implementation; within 2 months of sustainment start
Teamwork (Eff) Professionalism (Eff)	Strong teamwork and professionalism are expected to result from protocol use	Handoff-level; field notes from trained site-based staff (qual)	Quarterly, Years 2–5
Clinician satisfaction (Eff)	Clinician satisfaction is an early indicator of effectiveness	Clinician-level; surveys (quant); site visit findings (qual)	Annually, Years 1–5
Clinician workload (Eff)	Workload influences clinicians’ EBP use; fidelity is likelier if workload is unchanged or lower	Clinician-level; NASA Task Load Index [45] (quant); field notes, site visit findings (qual)	Quarterly, Years 2–5
Information omissions (Eff)	Per-protocol handoffs will show fewer information omissions	Handoff-level; direct observations* by trained site-based staff (quant)	Monthly, Years 2–5
Adverse events (Eff)	Per-protocol handoffs include enable the prevention of adverse events by promoting shared team understanding of patients’ care	ICU-level; composite measure based on 10 routinely collected measures of care (AHRQ PSI 90 [56])	Quarterly, Years 2–5

*AHRQ* Agency for Healthcare Research and Quality, *AIM* Acceptability of Intervention Measure [54], *Eff* effectiveness outcome, *FIM* feasibility of intervention measure [54], *IAM* intervention appropriateness measure [54], *ICU* intensive care unit, *Imp* implementation outcome, *PSI* patient safety indicator, *qual* qualitative measure, *quant* quantitative measure

We selected fidelity as our primary implementation outcome based on our original study, in which increased effectiveness of the HATRICC protocol, as measured by information completeness, was observed with increasing fidelity to the process [14]. Though the concept of handoff fidelity could be defined in different ways given that the OR to ICU handoff is a complex sociotechnical work system, we define fidelity as a count of adherence to the ten steps defined in the original process.

In selecting a primary effectiveness outcome, we considered outcomes most likely to be impacted by care decisions immediately following the handoff. In the hours following ICU admission, these decisions relate to maintenance of function of organs receiving most of the cardiac output and the organs most likely to be compromised by surgery and anesthesia. In practice, these organs are the brain, heart, lungs, kidneys, and blood. A composite measure is most appropriate given that handoff-related decisions may affect one or more of these organs. We also note that the use of composite outcomes is common in perioperative handoff research [57–61] owing to an improvement in power to detect associations with component outcomes that have low individual incidence. We selected a composite measure of organ failure as captured by perioperative patient safety indicators (PSIs) defined by the Agency for Healthcare Research and Quality (AHRQ) [53]. Our study hospitals routinely collect these PSIs for reporting to Vizient®, which means that our site teams will not have to undertake additional, potentially burdensome data collection to capture this outcome. We focused on those PSIs related to surgery and those most likely to be influenced by an OR-to-ICU handoff, based on our clinical experience with perioperative decision making.

Secondary implementation outcomes include feasibility, acceptability, appropriateness, sustainment, and affordability (i.e., implementation cost). Secondary effectiveness outcomes include teamwork, clinician satisfaction and workload, information omissions, and adverse events. We may opt to repeat interviews and focus groups if quantitative findings suggest problems with implementation or if implementation teams express concerns about the implementation process.

### Aim #4: design and create a digital toolkit for other ICUs to identify implementation determinants, customize an OR to ICU handoff protocol, and select appropriate implementation strategies

Using the lessons learned from customizing the HATRICC protocol and selecting and deploying various implementation strategies, we will create a digital toolkit for public use. A toolkit is a collection of information, resources, and details about how to implement a given process [62]. The creation of implementation toolkits is not uncommon [62–64], as they may help narrow the gap between implementation and practice. The web-based HATRICC-US toolkit will make the study findings usable by allowing users to input contextual factors, design and adapt process maps, select implementation strategies, and download customizable versions of OR to ICU handoff protocols. The goal of this toolkit is to provide smaller, less well-resourced, and community-based settings the opportunity to replicate aims 1 and 2 of the HATRICC-US study in silico, facilitating the uptake and use of standardized OR to ICU handoffs.

The HATRICC-US toolkit will be created using human-centered design (HCD), which incorporates the perspectives of current and potential stakeholders in creation of the toolkit to optimize usability [49, 65].

### Sample size calculation

Sample size will vary by aim and approach. In general, qualitative data will be sampled until meaning saturation [66] is reached. For our co-primary quantitative outcomes, additional detail is provided below. We assume a type 1 error rate of 0.05 despite having co-primary outcomes, as the implementation outcome of fidelity and the effectiveness outcome of new-onset organ failure are drawn from different analytic samples (handoff vs. ICU, respectively).

Co-primary outcome #1—Fidelity (patient handoff-level outcome): We selected a sample size sufficient to confer at least 90% power to detect a 1-point increase in fidelity; this corresponds to 15 handoffs per ICU per month, or 3–4 handoffs per ICU per week. The total sample for this outcome is expected to be between 1920 and 2880 handoffs.

Co-primary outcome #2—Number of new-onset organ failures (ICU-level outcome): The number of eligible patients is fixed given the focus on ICU-level outcomes. We determined that at the expected available sample size, we achieved > 90% power to observe at least a 10% relative decline (our threshold for the minimally important clinical difference [67]) in the number of new-onset organ failure cases, from 1.5 cases per 100 patients to 1.35 cases per 100 patients. The total expected patient count for this ICU-level outcome is between 20,000 and 30,000 patients.

### Data analysis

To analyze qualitative data, we shall use a mix of inductive and deductive approaches. The inductive approach will use applied thematic analysis [68], selected because we have a process-oriented goal—applying findings to implementation strategy selection. For our analysis, qualitative data will be coded in cycles by trained research assistants. The first cycle of coding will enable generation of a codebook grounded in the data. During successive coding cycles, we will refine the codebook to reflect emerging themes and recode data using a constant comparative approach [69]. The coding team will meet to refine the codebook and to explore emerging themes. For the deductive approach, we will compare our codebook to relevant constructs from the TICD Checklist [38] to look for similarities and differences. We will also examine how different levels of the social ecologic model [40] manifest themselves in the data. This mix of inductive and deductive approaches will allow us to discern findings that may not be reflected in the published literature while still allowing us to translate our findings to established behavior change theories. We will use the qualitative data analysis program NVivo (QSR International, Doncaster, Australia) to manage qualitative data.

The quantitative data from aim 1 are descriptive. We will therefore use descriptive statistics to summarize findings from observations and surveys with stakeholders during this contextual inquiry phase. We will develop visual and mathematical summaries of overlap and concordance, such as Venn diagrams, influence diagrams, and clustering analyses to facilitate understanding of our findings and to communicate these findings effectively with local implementation teams and other stakeholders [70, 71].

The aim 3 primary analysis will utilize a mixed-effects negative binomial regression model with random effects for ICU site and fixed effects for time to account for the stepped-wedge cluster randomized design. Based on the distribution of the data, we may perform sensitivity analyses using linear regression (for fidelity) and logistic regression (for new-onset organ failure).

## Discussion

HATRICC-US is a prospective multicenter Type 2 hybrid effectiveness-implementation mixed methods quasi-experimental study designed to test effectiveness and implementation outcomes after standardizing OR to ICU handoffs through use of a protocol. To our knowledge, this will be the largest and first multi-institutional study of OR to ICU handoffs. This study is innovative in at least two ways. First, we use engineering approaches (i.e., elicitation interviews, process mapping, participatory design, human centered design) to bolster contextual inquiry, identification of implementation determinants, and dissemination of study findings. We demonstrated in our pilot work that workflow considerations are paramount in the fast-paced ICU environment, and we believe that the use of engineering methods will increase the likelihood that clinicians will exhibit fidelity to OR-to-ICU handoff protocols.

Second, we focus on the implementation of a complex procedure in critical care, a setting that is under-represented in the implementation science literature. There are important reasons to believe that the critical care setting presents challenges to the application of findings derived from other settings. Specifically, the time scale over which decisions are made may be seconds to minutes, rather than hours to days or longer. Under such time pressure, individual clinician decision-making processes are likely to rely on intuition and naturalistic decision-making rather than deliberative thought [72, 73], which will influence the effectiveness of implementation efforts focused on individual behavior change. Additionally, critical care has a recent history of important reversals in evidence [74–76] leading to evidence skepticism among critical care practitioners that must be confronted in any implementation effort. Recent systematic reviews have shed light on some of the factors influencing EBP adoption and fidelity in critical care [8, 77], but these syntheses are limited by the studies that inform them, most of which were not grounded in theories relevant to implementation science. This project represents an important step forward in that it is grounded in implementation theory from the outset. By using this theory-based approach, we will inform future implementation science efforts in the critical care setting.

Despite the strengths of this study, it has limitations. First, this study takes place in academic medical centers in the USA. We selected these sites based on their ability to participate in a multicenter study, an expressed interest in improving OR to ICU handoffs, and because they have a relatively high volume of OR to ICU handoffs (as compared, for example, to dedicated medical ICUs). These decisions limit the transferability of findings, though we will detail contextual factors and implementation decisions in published reports. It is our hope that the digital toolkit will facilitate the uptake of research findings in a broader group of care settings. We aim to collect information from toolkit users, i.e., OR to ICU handoff stakeholders, that will enable us to adapt it for broader relevance. Second, though the intervention phase of the study will be almost 4 years long, sustainment data will be limited to just over 1 year for the last ICU to implement the new protocol. Third, the pragmatic nature of the study limits the ability to collect detailed information about relevant process measures, such as handoff accuracy. However, individual sites will be encouraged to conduct additional related studies that may provide deeper insight into implementation at the ICU level. Fourth, the implementation strategies used for each ICU are likely to be slightly different, limiting our ability to speak to the effectiveness of any given strategy. Despite these limitations, rich contextual information will be provided in published manuscripts to offer insights about the effectiveness of implementation strategies used. We will also explore the use of configurational comparative methods to better understand the contribution of individual implementation strategies to fidelity. Finally, there is a key tension between the idiographic (i.e., generalizable) and nomothetic (i.e., context-specific) aspects of this intervention that we may not be able to completely disentangle. Still, we anticipate being able to more clearly define the “core” versus “adaptable periphery” [78] of this intervention with this multicenter study in ways that are useful for other implementation efforts in critical care.

If successful, the HATRICC-US study will have integrated implementation science and engineering approaches like participatory design to customize and implement an evidence-based practice in the fast-paced critical care environment. This study is poised to shed light on effective approaches to implement complex sociotechnical interventions in acute care settings. Ultimately, this approach may prove useful in promoting the uptake and sustained use of proven-effective interventions in the intensive care unit.

## Supplementary Information

**Additional file 1.**

## Data Availability

At the time of manuscript submission, data collection from key informant elicitation interviews had commenced. Upon study completion, any datasets used and/or analyzed during the current study will be available from the corresponding author on reasonable request.

## Abbreviations

EBP: Evidence-based practice
EPIS: Exploration, Preparation, Implementation, Sustainment (model name)
HATRICC: Handoffs and Transitions in Critical Care (study name)
HATRICC-US: Handoffs and Transitions in Critical Care – Understanding Scalability (study name)
HCD: Human-centered design
ICU: Intensive care unit
IRB: Institutional review board
OR: Operating room
PI: Principal investigator
sIRB: Single institutional review board
SMART-IRB: Streamlined, Multisite, Accelerated Resources for Trials IRB Reliance
TICD: Tailored Implementation for Chronic Diseases (model name)
